# Post-COVID: a specific neuropsychological profile in performance deficits

**DOI:** 10.1007/s00702-026-03209-w

**Published:** 2026-06-29

**Authors:** Britta Müller, Ulrike Balz, Katharina Bartels, Farina Eggers, Micha Löbermann, Luise Nostitz, Juliane Schmidt, Carola Schwarz, Christine Wossidlo, Emil C. Reisinger, Peter Kropp

**Affiliations:** 1 Institute of Medical Psychology and Medical Sociology, Rostock University Medical Centre, Rostock, Germany; 2 Division of Tropical Medicine and Infectious Diseases, Department of Internal Medicine II, Rostock University Medical Center, Rostock, Germany

**Keywords:** Post COVID, Attention, Memory, Wxecutive functions, Mood

## Abstract

**Background:**

SARS-CoV-2 is frequently associated with cognitive impairment. When symptoms persist for more than four weeks after acute infection, a Long-COVID condition is diagnosed. When persisting more than 12 weeks, Post-COVID ist diagnosed. Cognitive complaints commonly include impairments in attention, memory, and executive functions. However, precise data on the specific pattern and extent of these deficits remain limited. Methods: Results: Conclusions: The findings indicate that attentional impairments are more prevalent than memory deficits in this Post-COVID cohort. Furthermore, distinct cognitive profiles can be identified using cluster analysis, ranging from globally impaired to cognitively preserved subgroups. These results may be relevant for clinical characterization and rehabilitation planning in Post-COVID conditions.

**Methods:**

We examined 88 patients (mean age 50.9 years, 80.7% female) diagnosed with COVID symptoms according to the WHO-criteria. Neuropsychological assessment was performed using a standardized test battery covering attentional performance, working memory, and executive functions. A cluster analysis was conducted to identify subgroups based on cognitive performance profiles.

**Results:**

Overall, 55.7% of patients showed below-average attentional performance, whereas memory deficits were less frequent (33.0%). Impairments in executive functions were similarly distributed across the sample. Cluster analysis yielded a three-cluster solution. Cluster 1 (34.1%) was characterized by pronounced impairments across all cognitive domains. Cluster 2 (20.0%) showed generally below-average cognitive performance; however, verbal fluency was preserved at or above average in all participants. In Cluster 3 (17.6%) more than 50% of patients in performed within average to above-average ranges across all cognitive domains.

**Conclusions:**

The findings indicate that attentional impairments are more prevalent than memory deficits in this Post-COVID cohort. Furthermore, distinct cognitive profiles can be identified using cluster analysis, ranging from globally impaired to cognitively preserved subgroups. These results may be relevant for clinical characterization and rehabilitation planning in Post-COVID conditions.

## Introduction

Severe Acute Respiratory Syndrome Coronavirus Type 2 (SARS-CoV-2) was first identified at the end of 2019 and is the cause of the global COVID-19 pandemic. The infection can cause systemic multi-organ disease, which is associated with a wide range of clinical manifestations and leads to long-term health impairments in some of those affected. If symptoms that cannot be explained by an alternative diagnosis persist for more than four weeks after the acute infection, this is referred to as “long COVID” or “post-acute sequelae of COVID-19” (PASC) according to the definition of the British National Institute for Health and Care Excellence (Dietz et al. 2024; National Institute for Health and Care Excellence (NICE), 2024). Post-COVID syndrome (PCS) is defined as lasting twelve weeks or longer and thus represents a specific phase within the Long- COVID spectrum (Nalbandian et al. 2021). According to data from a large British sample of children and adults from England and Scotland in 2024, the prevalence of Post-COVID (symptoms ≥ 12 weeks after acute COVID-19 infection) was 41.32% (95% CI: 39.27–43.37 (Abd Razak et al. 2024). People aged 35–65 were most affected. Numerous studies have demonstrated a significant impact of symptoms on the ability to work (Gandjour 2023; Reuschke and Houston, 2023). Known risk factors for Post-COVID include female gender, a high number of different symptoms during acute COVID disease, infection with early SARS-CoV-2 variants, and lack of prior vaccination against SARS-CoV-2 prior to the actual infection. The Long- and Post-COVID syndromes are characterized by pronounced fatigue and cognitive difficulties during the acute infection and for a prolonged period thereafter. For this reason, involvement of the central nervous system is assumed (Chollet y Leger, 2023). In many studies, cognitive deficits are the second most common symptom after fatigue (Cirulli et al. 2020; Davis et al. 2021). They occur in about 70% of Post-COVID patients (Cirulli et al. 2020). Cognitive deficits are particularly evident in the areas of both attention, executive functions and memory and are often accompanied by anxiety, depressive symptoms, and fatigue (Ceban et al. 2022; Nicotra et al. 2023). Therefore neuropsychological diagnostics should be used to quantify cognitive deficits (Lemhöfer et al. 2024). In the area of attention, the subfunctions intrinsic alertness, sustained attention, focused attention, and behavioral inhibition are often described being affected (Negrini and Schmidt, 2023). Memory deficits are observed in the short-term area, in spatial working memory, and in delayed recall, indicating a disorder in the medial temporal lobe and hippocampus (Hampshire et al. 2024). The lack of treatment options at the beginning of the pandemic plays an important role. Additionally the duration of the disease and hospitalization due to SARS-Cov-2 are also decisive factors for more severe symptoms. Severe symptoms are often characterized by both inflammatory and atrophic processes in the memory regions being assumed (Hampshire et al. 2024). While Post-COVID has now been studied in large cohorts (Cheetham et al. 2023; Liu et al. 2022; Zhao et al. 2023) there is a lack of precise data on the actual and specific cognitive performance deficits. In particular, the question arises as to whether cognitive impairments are more of a memory or more an attention problem. The aim of the present study is to close this research gap by, first, describing the cognitive performance deficits in adult individuals affected by Post-COVID and relating them to the corresponding age norms, taking into account psychological symptoms (depressive and anxiety symptoms); second, identifying predictors of cognitive performance deficits and, thirdly, weighing up attention and memory disorders.

## Methods

### Sample

The sample was recruited consecutively. Patients who presented at the Post-COVID outpatient clinic (Department of Internal Medicine / Division of Tropical Medicine and Infectious Diseases, University Medical Center Rostock), which opened in 2022, were informed about the study’s objectives by the attending physicians. The time interval between acute COVID-19 – condition and our examination ranged from 5 to 26 months (mean 14 months). Written informed consent was required for participation in the study. Inclusion criteria were age ≥ 18 years, previous acute COVID-19 infection, and self-reported Post-COVID symptoms. All participants fulfilled the WHO-criteria for a Post-COVID condition, defined as symptoms persisting for at least 3 months after acute infection and described by Soriano et al. (2022). Participants did not receive any medication with impact on fatigue, attention, or other neuropsychological variables.

### Data acquisition

The neuropsychological examination was conducted by clinical psychologists and lasted between 90 and 150 min. A medical history was taken to record subjective cognitive complaints. This was followed by cognitive testing to assess attentional parameters (general responsiveness and divided attention), memory (working memory, verbal learning, and memory performance), and executive functions (impulse control, adaptability, and divergent thinking). This test scheme has proven itself in other studies (Seibert et al. 2025). In addition, intelligence levels and word fluency were assessed. The test results were expressed in percentile values based on the respective normative test data (corrected for age, educational level, and/or gender).

The individual areas are listed below. See also Table 2 for a an overview.

#### Attention

In the area of attention, reaction speed, reaction alertness and divided attention were assessed using the test battery for attention testing (TAP 2.3.1, Zimmermann, Fimm, 2019) with the subtests Alertness (with and without acoustic warning tone) and Divided Attention. The TAP is a computer-based neuropsychological test procedure that measures various components of attention with good to very good parameters of reliability and validity (manual). The median reaction time as an indicator of general reaction speed, the standard deviation of the median as an indicator of performance stability, the characteristic score of phasic alertness, and the omission rate in divided attention as an indicator of attention capacity are examined here.

#### Memory

In the area of memory, the TAP working memory subtest was performed in the “2-back paradigm” to assess working memory. In this test, patients had to simultaneously store, update, and compare information. The test examined omissions as an indicator of a lack of control in the flow of information, as well as the error rate as an indicator of moments of inattention (Fimm 2012). Furthermore, verbal learning performance, consolidation, and recognition performance were assessed according to the manual using the Verbal Learning and Memory Test (VLMT, Helmstaedter et al. 2001). To assess consolidation performance, a free recall of previously learned words was conducted after 20 to 30 min, followed by a recognition test in which the patient was asked to indicate which words from the originally learned word list they remembered by answering yes or no to a list of words read aloud. Overall learning, consolidation, and recognition performance are examined. The VLMT shows high scores in the test quality criteria (Helmstaedter et al. 2001).

#### Executive functions

To assess executive functions, the standardized TAP with the Go/Nogo and Flexibility subtests was also used to make statements about impulse control and cognitive flexibility. The error rate was examined as an indicator of the extent of impulse control, and the median reaction time as an indicator of the speed of the decision-making process (Zimmermann, Fimm, 2019).

The Regensburg Word Fluency Test (RWT, Aschenbrenner et al. 2000) was used to measure formal lexical and semantic word fluency with four subtests. The test was conducted strictly according to the manual with the four subtests. This allows word fluency performance in the formal lexical and semantic areas to be assessed. The RWT is suitable due to its good validity and reliability (Aschenbrenner et al. 2000).

#### Intelligence

The multiple-choice vocabulary intelligence test (MWT-A, Lehrl, 2024) was used to assess the participant’s vocabulary and associated verbal abilities, which enable an estimation of their premorbid intelligence level.

The test procedures used are listed in the table below.

In addition, self-report questionnaires were used to collect data on other physical (Fatigue Assessment Scale, FAS, Michielsen et al. 2003) and psychological symptoms, especially life quality (SF12, Morfeld et al. 2011), depression (Beck Depression Inventory BDI-II, Hautzinger et al. 2006).

**Table 1:** Compilation of the standardized testing procedures used

Area	Basic function	neuropsychological subtest
Attention	Reaction time/ alertness	TAP – Alertness
	Divided attention	TAP – divided attention
Memory	Working memory	TAP – working memory
	Verbal learning	VLMT – ∑dg1-5
	Consolidation	VLMT – dg5-7
	Recall	VLMT – W-F
Executive functions	Impulsecontrol	TAP – Go /Nogo
	Cognitive flexibility	TAP- flexibility (change, verbal)
	Divergent thinking	RWT – formallexical fluency (U1, U2); semantic fluency (U3,U4)
Intellectual functions	General intelligence level	MWT-A

TAP: Test for attentional performance; VLMT: verbal learning and memory test; RWT: Regensburg word fluency test; MWT-A: Cristallized intellectual function test, version A; dg – round 5–7, U: subtests (U1 – m-words, U2 – alternate H-T, U3-food, U4-alternate flower-clothes

#### Fatigue

The survey was conducted using the Fatigue Assessment Scale (FAS, Michielsen et al. 2003), consisting of 10 items to be rated on a five-point Likert scale (1 = never, 5 = always). The higher the score, the more pronounced the fatigue. A score of more than 22 points is considered moderate fatigue, while a score of more than 34 points is considered very severe fatigue. The FAS and BDI-II questionnaires were given to the patients to fill out at home. They were then returned by mail.

#### Depressive symptoms

BDI-II: The Beck Depression Inventory - BDI-II (German edition by Hautzinger et al. 2023) is a self-assessment tool used to determine the severity of depressive symptoms. It includes 21 items with a four-point scale (0 to 3) that are formulated depending on the symptoms inquired about, with a higher score indicating more pronounced symptoms. Total scores are calculated and corresponding cut-off values provide information about the severity of the symptoms. A score of 13 points or less indicates no evidence of clinically relevant depression. A score of 14 to 19 points indicates mild symptoms, 20 to 28 points indicates moderate symptoms, and 29 points or above indicates severe depressive symptoms.

**Fig. 1:**
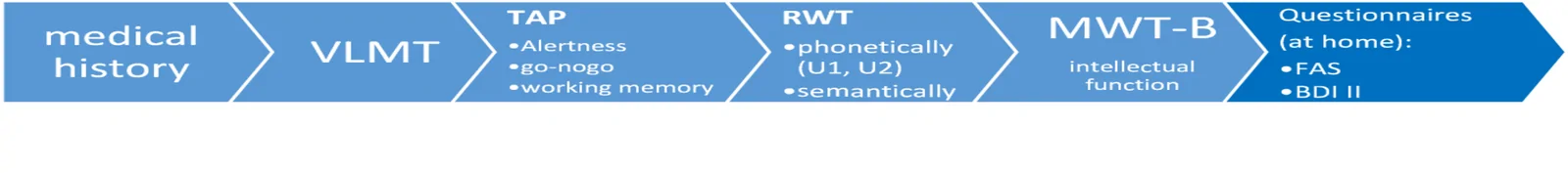
Chronological sequence of test diagnostics. Abbreviations are explained in the text

The chronological sequence of the test diagnostics can be seen in Fig. 1:

### Data reduction

Standardized data were calculated and qualified into “average, below average, and above average”. The average range is between 90 and 110 for standard values (SV), 85 and 115 for IQ-values (IQ), and between 40 and 60 for T-values. Descriptives were calculated according to their amout of one of these classes.

A Two-Step Cluster Analysis was conducted to identify distinct groups based on six dichotomized variable. All variables were coded as 0 = absence and 1 = presence of each dichotomized variable. Prior to clustering, multicollinearity was assessed and found not to be an issue, with all variance inflation factor (VIF) values ranging between 1.2 and 2.2. The analysis was performed using SPSS version 26. The log-likelihood distance measure was employed, which is appropriate for categorical variables, and the number of clusters was pre-specified as three based on theoretical considerations and interpretability. The Two-Step algorithm first constructed a Cluster Features (CF) tree to pre-cluster cases and then applied hierarchical clustering to merge pre-clusters into the final clusters. Cluster quality was evaluated using the Silhouette measure of cohesion and separation, with values above 0.5 considered indicative of reasonable cluster separation. The interpretability and plausibility of the clusters were confirmed by examining the distribution of the input variables across clusters. Cluster membership probabilities were also inspected to ensure robust classification. This procedure allowed for the identification of distinct, interpretable subgroups within the sample, while accounting for the categorical nature of the input variables and ensuring minimal multicollinearity among predictors.

## Results

### Sample

The sample characteristics are shown in Table 2. Of the 88 individuals, 71 (80.7%) were female. The average age was 50.9 years (SD = 10.7), ranging from 26 to 75 years. In terms of education, 35 people (39.8%) stated that their highest educational qualification was a (specialized) high school diploma, 49 people (55.7%) had completed secondary school, and two people (2.3%) had a secondary school diploma or no school diploma. With regard to psychological symptoms, we focused on depressive symptoms. Here, 54 of the 88 participants (61.4%) reported at least mild depressive symptoms in the BDI-II. All participants showed at least mild to moderate fatigue with a score of 22 points or higher on the FAS (Fatique Assessment Scale, Vries et al. 2004). Almost three-quarters of the participants (70.5%) even reported severe fatigue with a score of more than 34 points. The general intelligence level determined using the MWT-A averaged an IQ score of 111.7 points (SD = 14.6), ranging from 85 to 145 IQ points. Median-time between begin of SARS-CoV-2 –infection and neuropsychological tests ranged between 5 and 26 months with a mean of 14 months.

**Table 2:** Description of the sample based on age, sex, education, and COVID-19 symptoms

Variable		Total Sample *N* = 88	Male *n* = 18	Female *n* = 70
Age, mean (SD)		50.9 (10.7)	50.7 (13.8)	50.9 (10.0)
	Missing	0	0	0
Education (ISCED), n (%)	Low (Level 1/2)	4 (4.6)	1 (5.9)	3 (4.2)
	Intermediate (Level 3/ 4)	49 (55.6)	7 (41.2)	42 (59.2)
	High (Level 5/6)	35 (39.8)	9 (52.9)	26 (36.6)
	Missing	0	0	0
Severity of COVID-19 symptoms, n (%)	Mild	75 (84.3)	16 (88.9)	59 (84.3)
	Moderate	11 (12.5)	1 (5.6)	10 (14.3)
	Severe	2 (2.3)	1 (5.6)	1 (1.4)
	Missing	0	0	0
Post-exertional Malaise, n (%)		71 (80.0%)	16 (88.0%)	55 (78.0%)
	Missing	0	0	0
Dizziness, n (%)		49 (55.6%)	10 (55.6%)	39 (55.7%
	Missing	0	0	0
Headache, n (%)		62 (70.5%)	12 (66.7%)	50 (71.4%)
	Missing	0	0	0
Fatigue (FAS), n (%)	No (0–22)	2 (2.3)	0 (0.0)	2 (2.9)
	Moderate (23–34)	22 (25.6)	8 (47.1)	14 (20.3)
	Severe (> 34)	62 (72.1)	9 (52.9)	53 (76.8)
	Missing	2	1	1
Depression (BDI), n (%)	No (0–13)	31 (36.5)	8 (47.1)	23 (33.8)
	Mild (14–19)	20 (23.5)	4 (23.5)	16 (23.5)
	Moderate (20–28)	14 (16.5)	2 (11.8)	12 (17.6)
	Severe (> 28)	20 (23.5)	3 (17.6)	17 (25.0)
	Missing	3	1	2
General Intelligence (MWT-A), Mean (SD)		111.7 (14.6)	107.7 (11.9)	112.7 (15.1)
	Missing	1	1	0

*ISCED* International Standard Classification of Education, *FAS* Fatigue Assessment Scale, *BDI* Beck Depression Inventory, *MWT-A* Multiple-Choice Vocabulary Intelligence Test A, *SD* standard deviation

### Cognitive performance and psychological symptoms

#### Attentional performance

With regard to alertness (stimulus without warning signal), it can be seen that 55.7% of patients show a below-average reaction time on average, meaning that they need significantly longer time to react to stimuli than the normal sample. The standard deviation is also below average in 50.0% of patients, indicating a particularly high variability of reaction times. With regard to phasic alertness, i.e., the ability to increase attention in the short term, 27.3% of patients were below average.

When looking at divided attention, 45.5% of patients showed below-average performance, meaning they overlooked significantly more stimuli than the normal sample. All results are summarized in Table 3.

**Table 3:** Attentional performance

In %	TAP – Alertness			TAP – divided attention
	Median	SD	Phasic Alertness	Omissions
Below average	55.7	50.0	27.3	45.5
Average	44.9	45.5	50.0	46.6
Above average	0	4.5	22.7	6.8
Missing	0	0	0	1.1

*TAP* Test for attentional performance, *SD* standard deviation

#### Working memory

With regard to omissions, 30.7% of patients performed below average, and 29.5% performed below average in terms of errors, indicating a higher number of omissions and errors, respectively. In addition to the TAP, the VLMT was used to differentiate between verbal learning performance, consolidation, and recognition. This showed that 19.3% of patients performed below average in learning. In consolidation, 12.5% of patients performed below average, and in recognition, 25.0% performed below average. When looking at the z-standardized values of the VLMT, it was found that the patients performed within the average range: Learning: *M* = -0.16, *SD* = 0.93, Consolidation: *M* = -0.07, *SD* = 1.27, Recognition: *M* = -0.59, *SD* = 1.69. Results of working memory are summarized in Table 4.

**Table 4:** Working memory

In %	TAP – working memory		VLMT		
	Omissions	Mistakes	Learning	Consolidation	recall
Below average	30.7	29.5	19.3	12.5	25.0
Average	53.4	53.4	60.2	84.1	75.0
Above average	13.6	14.8	20.5	3.4	0
Missing	2.3	2.3	0	0	0

*TAP* Test for attentional performance, *SD* standard deviation, *VLMT* verbal learning and memory test

#### Executive functions

In terms of executive functions, 44.3% of patients showed below-average impulse control (see Table 4). Accordingly, patients showed longer reaction times than the standard sample. In addition, 19.3% of patients made more mistakes than the standard sample. In terms of cognitive flexibility, 22.7% of those affected performed below average in terms of errors, meaning they made more errors than the standard sample. In terms of overall flexibility performance, 29.5% of patients performed below average.

The RWT was used to test divergent thinking, which assessed formal lexical and semantic word fluency. It was found that 33.0% of patients performed below average in subtest 1 on formal lexical verbal fluency and 47.7% in subtest 2. In terms of semantic verbal fluency, 31.8% of patients were below average in subtest 3 and 27.3% in subtest 4 (see Tables 5 and 6).

**Table 5:** Executive functions - TAP

In %	TAP – Go/Nogo		TAP – Flexibility	
	Median	Mistakes	Mistakes	Overall
Below average	44.3	19.3	22.7	29.5
Average	45.5	80.7	52.3	51.1
Above average	10.2	-	23.9	18.2
Missing			1.1	1.1

TAP: Test for attentional performance; TAP-Go/Nogo – mistakes only two categories exist.

**Table 6:** Executive functions - RWT

In %	Formal lexical		Semantic	
	Subtest 1	Subtest 2	Subtest 3	Subtest 4
Below average	33.,0	47.7	31.8	27.3
Average	65.9	50.0	60.2	70.5
Above average	1.1	2.3	8.0	2.3
Missing	0	0	0	0

*RWT* Regensburg word fluency test

#### Identification of subgroups of cognitive impairments

A two-step cluster analysis was performed to identify subgroups of cognitive impairments. Six cognitive test variables were included in the analysis simultaneously in dichotomized form (0 = below-average performance, 1 = at least average performance) from the areas of “Reaction time/Alertness”, “Cognitive flexibility”, “Divided attention”, “Impulse control”, “Working memory”, and “Divergent thinking”. There was no multicollinearity; all variance inflation factor (VIF) values were between 1.2 and 2.2. The number of clusters was set at three. The clustering resulted in a solution with three clearly distinguishable groups (clusters 1–3). In addition, the analysis revealed a subgroup (28.2% of the sample) that clearly differed from clusters 1–3.

**Fig. 2:**
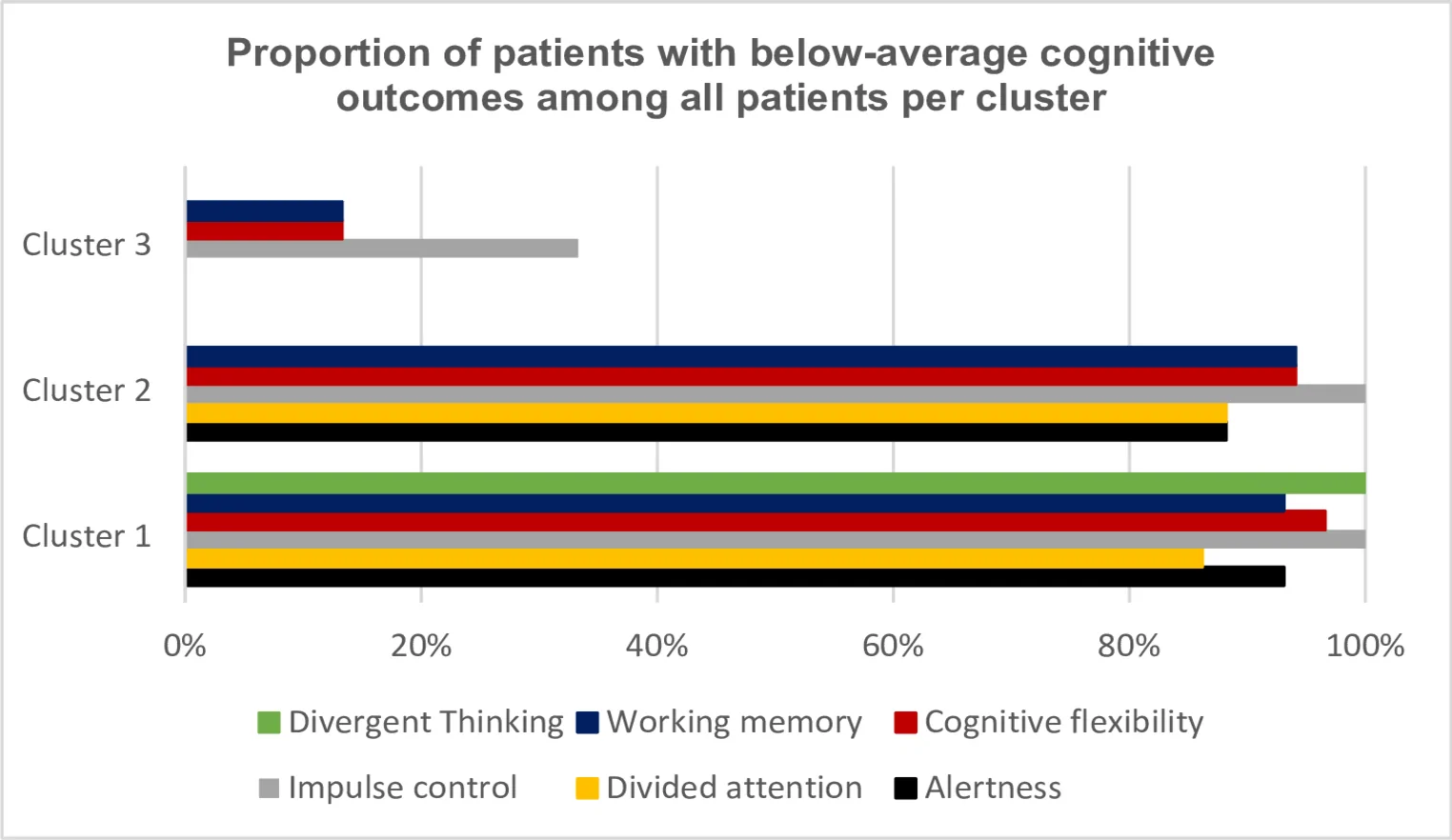
Composition of cluster 1–3 according to cognitive impairments

The silhouette index of the cluster solution was 0.7, indicating good cluster quality. 34.1% of participants formed cluster 1, 20.0% were in cluster 2, and 17.6% were in cluster 3. The three clusters differed significantly in their cognitive profiles (see Table 7). Cluster 1 was characterized by significant weaknesses in all cognitive areas. Cluster 2 also showed a high proportion of below-average performance, with one exception: in the area of verbal fluency, all participants performed at least at an average level. Cluster 3 was the highest-performing cluster; in all tests, the majority achieved at least average performance. The most significant input variable for the cluster analysis result was the word fluency test (predictor influence = 1.00). The influence of the other five predictors was at a comparable, medium level (0.60 to 0.72, see Fig. 2).

The subgroup included participants with very rare combinations. On the one hand, they showed good alertness and divided attention, but on the other hand, their impulse control, cognitive flexibility, working memory, and divergent thinking were predominantly impaired. One-third of them showed below-average performance in two, three, and four cognitive domains, respectively (see Table 7).

**Table 7:** Percentage of individuals with below-average performance in the clusters

Basic function (%)	Cluster 1 (*N* = 20)	Cluster 2 (*N* = 25)	Cluster 3 (*N* = 19)	Subgroup (*N* = 21)
Alertness	93.1	88.2	0	4.2
Divided attention	86.2	88.2	0	29.2
Impulse control	100.0	100.0	33.3	75.0
Cognitive flexibility	96.6	94.1	13.3	58.3
Working memory	93.1	94.1	13.3	58.3
Divergent Thinking	100.0	0	0	70.8

Additional correlation analyses revealed that in cluster 1, the proportion of individuals with a high school diploma and without depressive symptoms was above average. Cluster A includes an above-average number of individuals without a high school diploma and older than age of 50: Education: χ^2^(3,*N* = 85) = 12.41, *p* = .006; HADS-D: χ^2^(9,*N* = 85) = 19.69, *p*=.020; Alter: χ^2^(3,*N* = 85) = 8.49, *p* = .037).

## Discussion

Our findings are consistent with current research, which indicates that executive functions, attention, and processing speed are associated with Post-COVID (Panagea et al. 2025). However, our results show a more nuanced picture: comparatively severe impairments are evident particularly in the areas of alertness, divided attention, and inhibitory control (executive functions), while working memory and cognitive flexibility appear to be less affected. Furthermore, the current state of research is expanded to include the examination of the linguistic domain, which had not been sufficiently considered until now. Here, significant impairments are seen primarily in formal lexical word fluency, especially in subtest 2, in individuals affected by Post-COVID. The results of the cluster analysis indicate that the sample can be divided into four clearly distinguishable subgroups of cognitive performance profiles. These clusters differ not only in terms of overall performance level, but also in terms of specific patterns of strengths and weaknesses. Cluster 1 shows overall performance deficits. Cluster 2 shows an intermediate profile: pronounced weaknesses are offset by good verbal fluency. Cluster 3 can be interpreted as a high-performing group. The subgroup shows discrepancies between good alertness/divided attention vs. executive functions, flexibility, working memory, and verbal fluency, with no homogeneous pattern of characteristic combinations emerging.

In our study, impairments were primarily observed in attentional functions and specific aspects of executive functioning. Structural MRI studies have identified alterations in hippocampal subfields in Post-COVID populations, with associations to symptom severity (Thapaliya et al. 2025; Klein et al. 2021). However, these findings do not directly translate into measured memory impairments, and in our cohort, we did not observe evidence of memory deficits. This pattern may suggest that, in our sample, Post-COVID is more strongly associated with impairments in attentional functions rather than memory processes. This interpretation is consistent with findings by Hauke-Gleißner et al. (2025), who reported prominent abnormalities in children and adolescents, particularly in loss of motivation (72.9%), concentration and attention deficits (71.8%), worsened mood (53%), and increased anxiety (31.8%). Hampshire et al. (2024) could show that memory, reasoning, and executive function tasks were associated with the largest deficits compared to healthy controls. Using a comparable computerized assessment, they found that cognitive deficits were most evident in the domain of sustained attention, with patients showing slower reaction times and more omissions than expected compared with the Gaussian distribution.

This could be an indicator of a premature fatigue during the examination but not a genuine weakness in memory functions. The broader range of neurocognitive deficits, described elsewhere (Miller et al. 2025; Troitskaya et al. 2022), could not be confirmed. We therefore assume that Post-COVID symptoms cause a particular impact on attention functions due to a slower way of working and reduced motivational enhancement. This could also be the reason why Post-COVID patients show difficulties in memory tests. Often tests are also evaluated using reaction time analyses, resulting in incorrect error attribution. Cluster 2 and the subgroupin particular exhibit specific cognitive weaknesses in key areas, which may indicate different neurocognitive mechanisms. The findings contribute to the literature by showing that heterogeneous performance profiles within clinical populations should be taken into account. In practical terms, these results can help to develop tailored support or intervention programs. In sum our findings confirm that the performance impairments in Post-COVID patients are more likely to be attributed to a slower way of working with the result of a prolonged reaction time, and less to a problem with one of the measured memory functions. Thus, the problems therefore lie less in hippocampal function than in attentional activation.

Our study has several limitations. The sample size is small compared to other studies, and the results could have been even more striking with a larger sample. In addition, this study uses a cross-sectional approach, so no statement can be made about the course of the disorders. Since we did not take the duration of the illness into account, no statement can be made about the dynamics of the disorders. The duration of the examinations may have caused fatigue in the patients, which could have affected the results. Although fatigue was highly prevalent in the sample, its association with cognitive performance was restricted to three of 17 neuropsychological measures. However, subtle effects of fatigue-related fluctuations in attention on other cognitive domains cannot be excluded. Finally, the cross-sectional design limits causal inferences regarding the relationship between fatigue, attention, and cognitive performance. A further limitation of the present study is the lack of systematically collected longitudinal data on symptom progression across different phases of COVID-19 and Post-COVID illness. In particular, we were unable to reconstruct symptom trajectories from the acute infection phase through the post-acute period to the onset and persistence of Post-COVID symptoms. Future prospective longitudinal studies are therefore needed to better elucidate symptom trajectories and their potential relevance for the development of neuropsychological impairments in Post-COVID. We are also unable to make any statements about structural changes in the brain caused by COVID-19 infection. This would require postmortem analyses, as were already described by Riederer and Ter Meulen (2020). The strengths of the study lie in its differentiated presentation of cognitive abnormalities in Post-COVID, particularly with regard to the different effects on speed-related functions and memory functions. In addition, cluster analysis can be used to identify very different groups with different impairments. This would also allow appropriate interventions to be carried out.

## Data Availability

The data that support the findings of this study are not openly available due to reasons of sensitivity and are available from the corresponding author upon reasonable request. Data are located in controlled access data storage at Rostock University Medical Center.
